# Supplementary material for: Two classes of DNA gyrase inhibitors elicit distinct evolutionary trajectories toward resistance in gram-negative pathogens
Source: NPJ Antimicrob Resist. 2024 Mar 2;2:5. doi: 10.1038/s44259-024-00021-y (PMC11702832; doi:10.1038/s44259-024-00021-y)
Supplement: Supplementary file 1 — Supplementary Information [file 44259_2024_21_MOESM1_ESM.pdf]

Supplementary information

Two Classes of DNA Gyrase Inhibitors Elicit Distinct Evolutionary Trajectories Toward Resistance in Gram-Negative Pathogens

Semen A. Leyn[a†], James E. Kent[a†], Jaime E. Zlamal[a], Marinela L. Elane[a], Maarten Vercruysse[b], Andrei L. Osterman[a]

[a] Sanford Burnham Prebys Medical Discovery Institute, La Jolla, California, USA

[b] Roche Pharma Research and Early Development, Immunology, Inflammation, and Infectious Diseases, Basel, Switzerland

[†] Semen A. Leyn and James E. Kent contributed equally to this work.

Table of Content

I. SUPPLEMENTARY FIGURES 2

SUPPLEMENTARY FIGURE S1. 2

SUPPLEMENTARY FIGURE S2. 3

SUPPLEMENTARY FIGURE S3. 5

SUPPLEMENTARY FIGURE S4. 10

SUPPLEMENTARY FIGURE S5. 13

II. SUPPLEMENTARY METHODS 14

MORBIDOSTAT SETUP AND PROGRAMMING 14

SEQUENCING DATA ANALYSIS, VARIANT CALLING AND RANKING 15

POPULATION WGS DATA DECONVOLUTION FOR EVOLUTIONARY DYNAMICS AND CLONAL ANALYSIS 16

RNA ISOLATION AND RT-QPCR 17

REFERENCES FOR THIS DOCUMENT 19

I. Supplementary Figures

Supplementary Figure S1.

General workflow diagram of morbidostat-based evolution of resistance to GP6 compound in *E. coli* BW25113 and *A. baumannii* ATCC17978

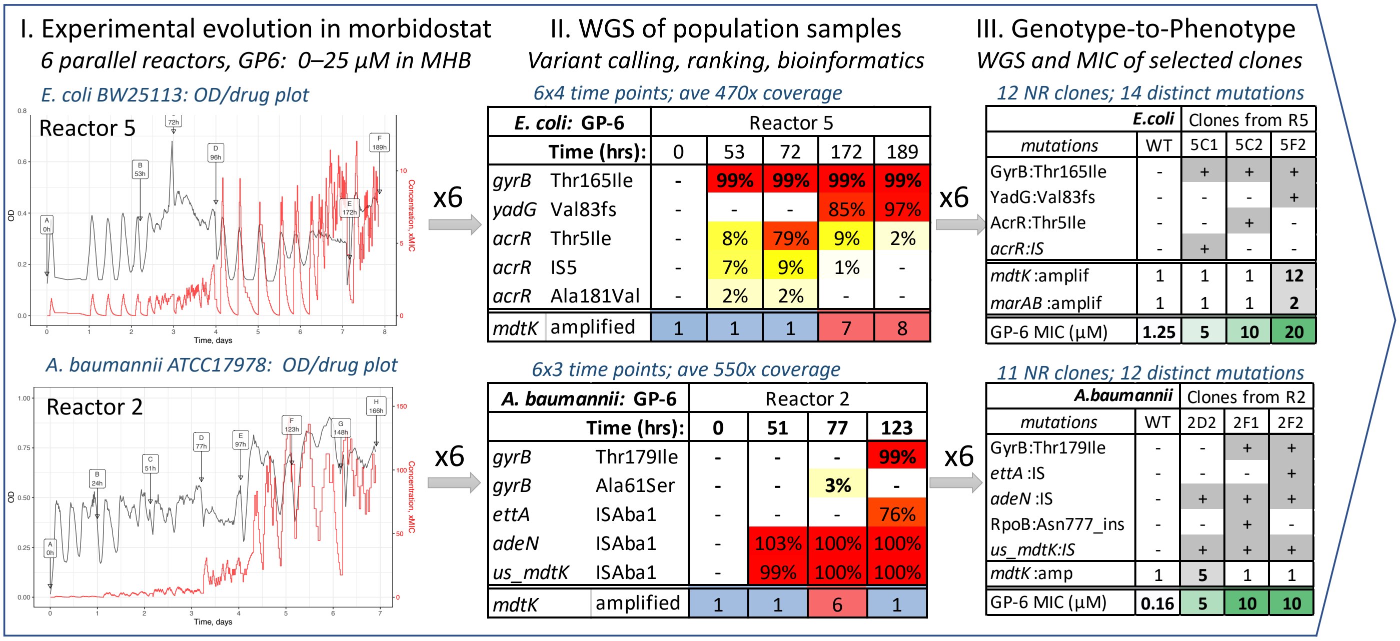

E. coli: GP-6		Reactor 5				
	Time (hrs):	0	53	72	172	189
gyrB	Thr165Ile	-	99%	99%	99%	99%
yadG	Val83fs	-	-	-	85%	97%
acrR	Thr5Ile	-	8%	79%	9%	2%
acrR	IS5	-	7%	9%	1%	-
acrR	Ala181Val	-	2%	2%	-	-
mdtK	amplified	1	1	1	7	8

A. baumannii: GP-6		Reactor 2			
	Time (hrs):	0	51	77	123
gyrB	Thr179Ile	-	-	-	99%
gyrB	Ala61Ser	-	-	3%	-
ettA	ISAba1	-	-	-	76%
adeN	ISAba1	-	103%	100%	100%
us_mdtK	ISAba1	-	99%	100%	100%
mdtK	amplified	1	1	6	1

E.coli		Clones from R5		
mutations	WT	5C1	5C2	5F2
GyrB:Thr165Ile	-	+	+	+
YadG:Val83fs	-	-	-	+
AcrR:Thr5Ile	-	-	+	-
acrR:IS	-	+	-	-
mdtK :amplif	1	1	1	**12**
marAB :amplif	1	1	1	**2**
GP-6 MIC (µM)	**1.25**	**5**	**10**	**20**

A.baumannii		Clones from R2		
mutations	WT	2D2	2F1	2F2
GyrB:Thr179Ile	-	-	+	+
ettA :IS	-	-	-	+
adeN :IS	-	+	+	+
RpoB:Asn777_ins	-	-	+	-
us_mdtK:IS	-	+	+	+
mdtK :amp	1	**5**	1	1
GP-6 MIC (µM)	**0.16**	**5**	**10**	**10**

Supplementary figure S2.

A: OD profiles (black line) and calculated drug concentration (red line) profiles for experimental evolution in morbidostat of *E. coli*. Collected samples are shown by arrows and labeled with time.

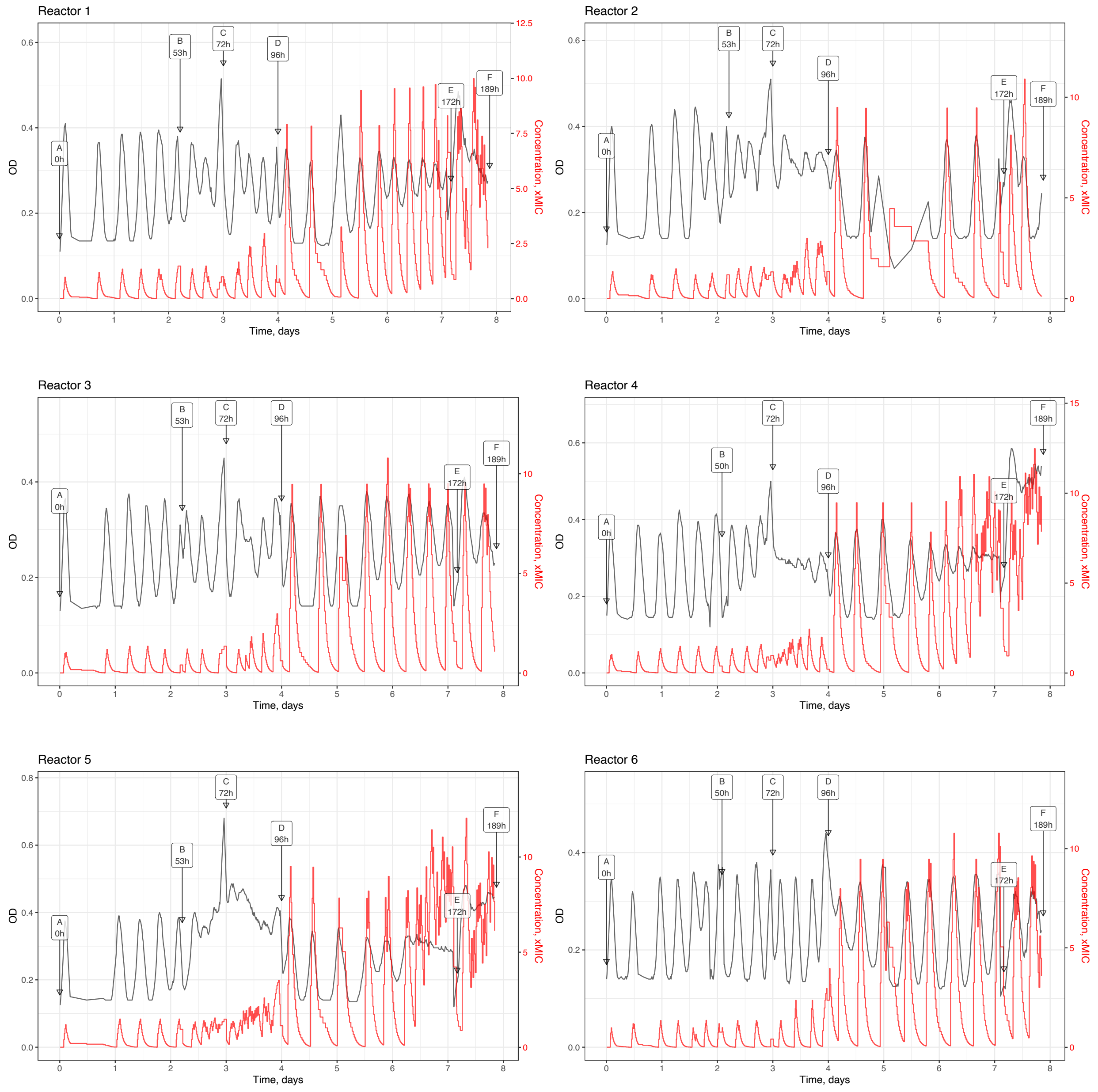

Supplementary figure S2 (continued)

B: OD profiles (black line) and calculated drug concentration (red line) profiles for experimental evolution in morbidostat of A. baumannii. Collected samples are shown by arrows and labeled with time.

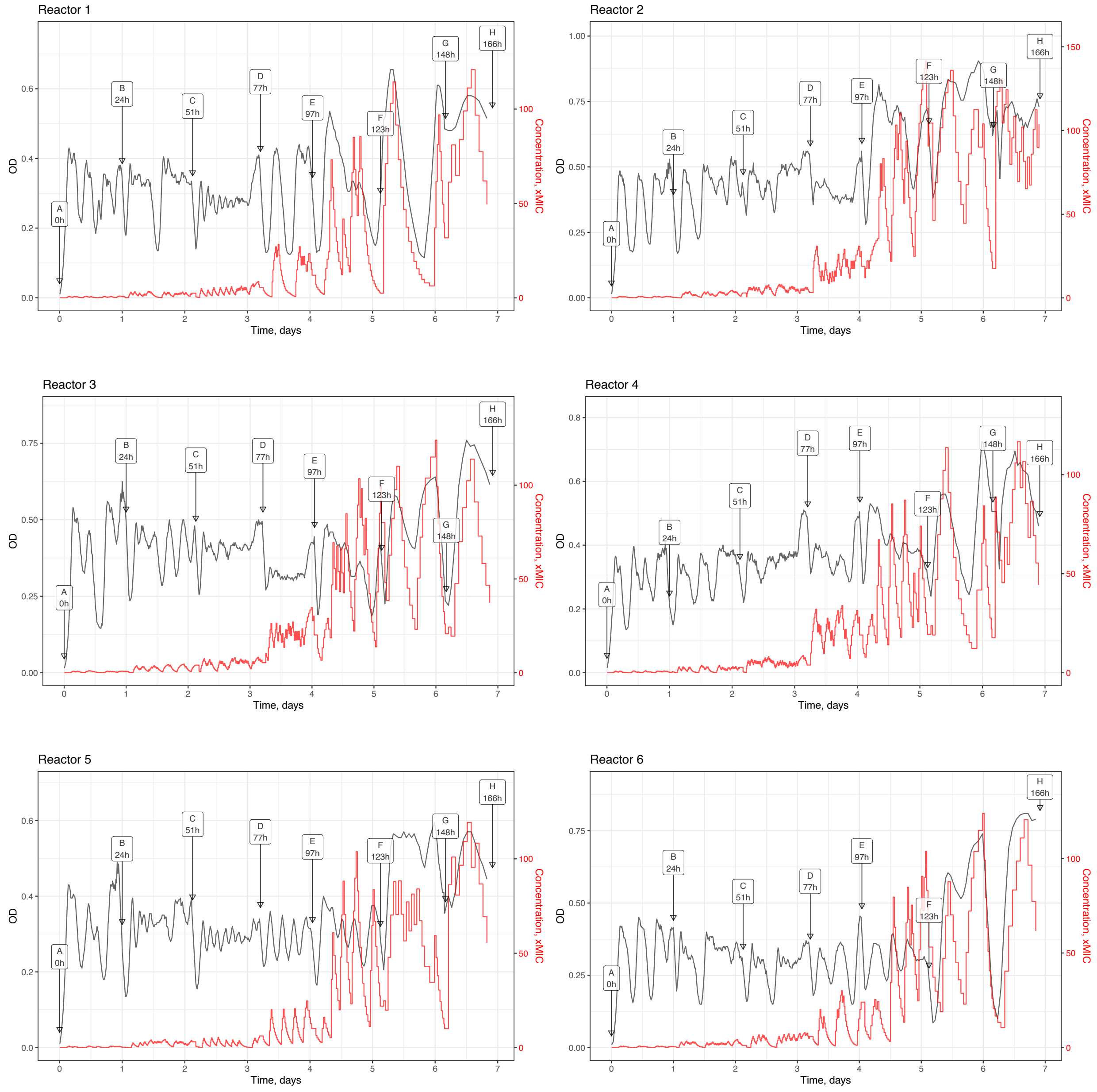

Supplementary Figure S3. Dynamics of major mutational variants in morbidostat-based evolution of resistance to GP6 compound in *E. coli* BW25113 (A) and *A. baumannii* ATCC17978 (B).

Supplementary Figure S3A

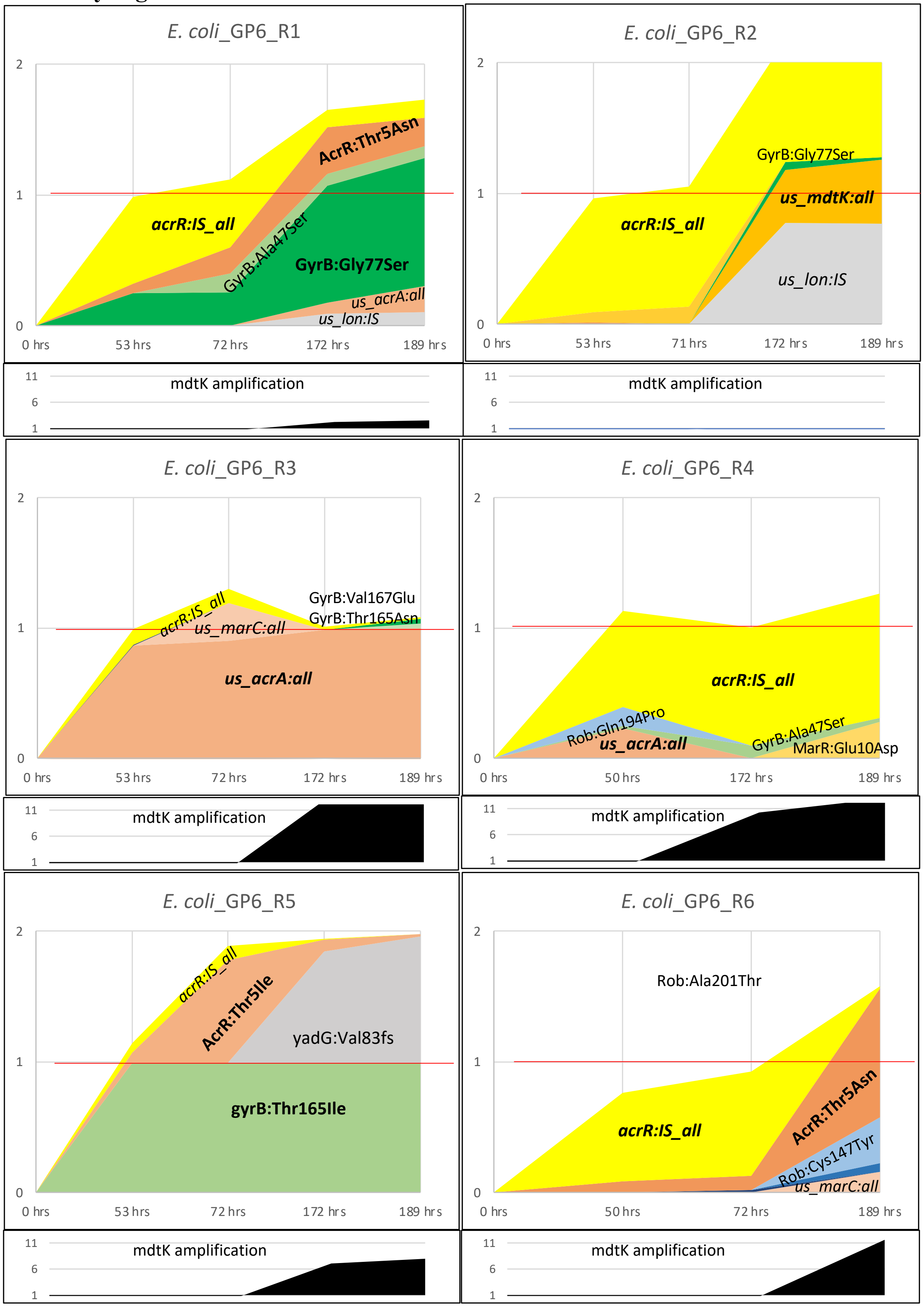

Supplementary Figure S3B

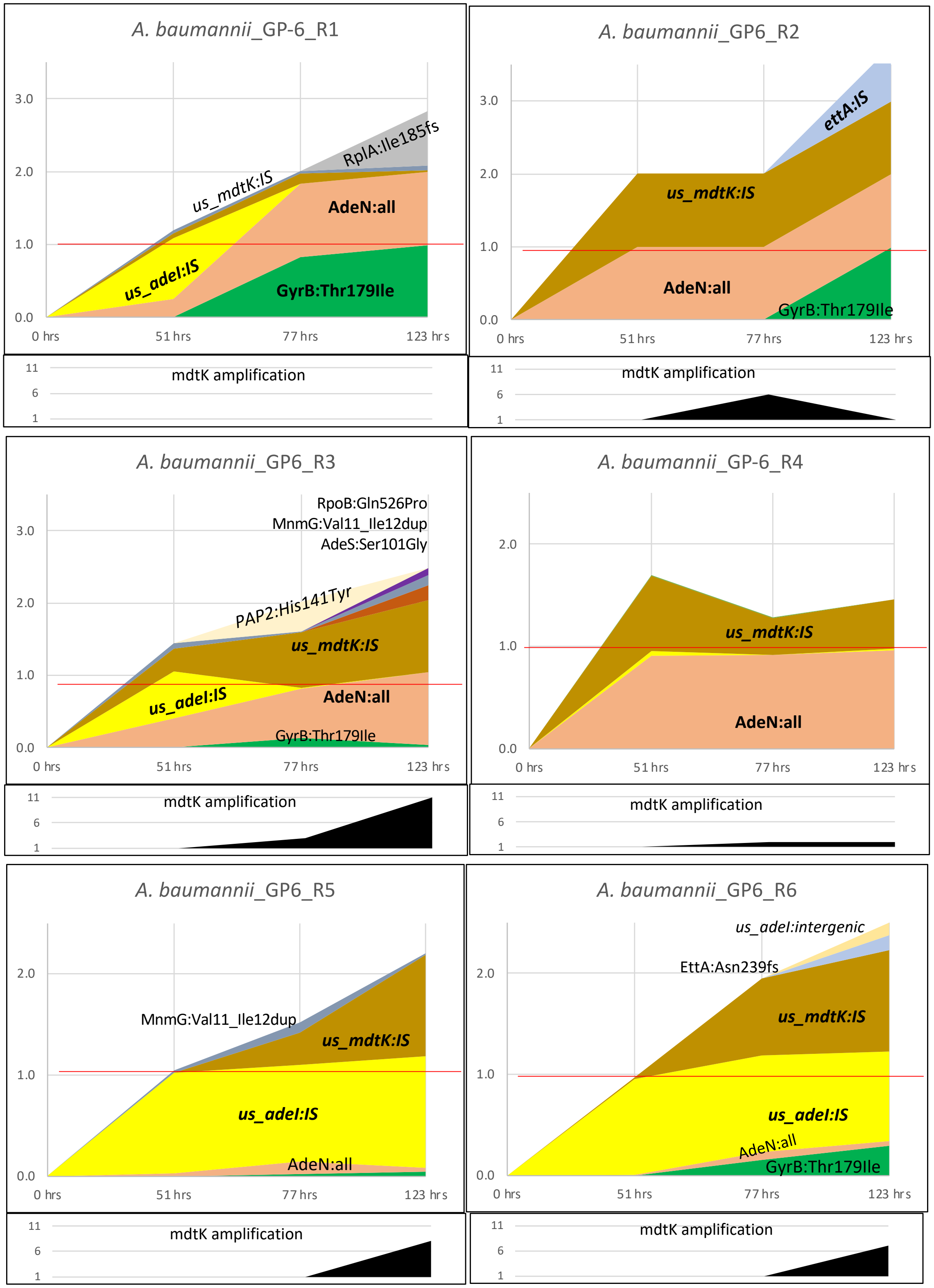

Cumulative area plots are shown for the most prominent mutational variants reaching relative abundance Amax ≥10% in at least one sample. Plots are shown for each of the six reactors R1-R6. The variant abundance data (from Supplementary Table S1 A and B) corresponding to each of the 6

reactors are plotted as the average number of corresponding mutations per cell (Y-axis) in the population vs time (X-axis). The red line (at average 1 mutation/genome) helps to emphasize the emergence of double and triple mutants. For each reactor, the upper plot shows relative abundances of SNVs and IS insertions. The lower plot shows the observed extent of amplification of MdtK locus, for which the relative abundance cannot be assessed in population WGS data.

Gene names and corresponding functions are explained in the legend to Table 1 (and in Supplementary Table S1A with more details and tabular data). A comparative analysis of these plots, which was only briefly described in the respective sub-sections of Results and Discussion, yields the following additional observations:

Drug target modification

In E. coli (Figure S3A):

GyrB: Among the 6 variants spanning 5 amino acid residues in *E. coli* GyrB, each of the two most prominent variants, GyrB:Thr165Ile and GyrB:Gly77Ser, dominated in one of the reactors reaching A_{max}=98-99%. The other three variants, GyrB:Thr165Asn, GyrB:Val167Glu and GyrB:Ala47Ser were less frequent reaching A_{max} in the range of 4-14%. The only variant localized remotely from the ATP-binding site, GyrB:Cys476Phe, showed the lowest A_{max}=2%, and its relevance for GP-6 resistance remains questionable.

In A. baumannii (Figure S3B):

GyrB: Of the two detected variants of *A. baumannii* GyrB, the most prominent, GyrB:Thr179Ile (a full equivalent of *E.coli* GyrB:Thr165Ile), emerged in 5 out of 6 bioreactors reaching 99% abundance in two of them. The second minor variant, GyrB: Ala61Ser (matching *E.coli* GyrB:Ala47Ser), appeared only in 1 reactor reaching A_{max}=3% prior to being competed out by the GyrB:Thr179Ile variant.

Efflux deregulation

In E. coli (Figure S3A):

AcrR: In reactors R1 and R6, the aggregated abundance of AcrR:IS variants peaked at 54 and 75%, respectively, at an earlier stage of evolution while decreasing (down to 14% and 1%) by the end of the run. In one of them (R6), the AcrR:Thr5Asn variant emerged at the later stage competing out an earlier dominant AcrR:IS variant. Overall, missense mutations in the same position (AcrR:Thr5Asn and AcrR:Thr5Ile) emerged independently in three bioreactors (R1, R5, R6), reaching A_{max}~36-99%. Quite likely, missense mutations at Thr5 position (in the DNA binding domain of AcrR) also lead to at least partial loss of AcrR function as a repressor of *acrR<>acrAB* divergon. Finally, four distinct SNVs were detected in the intergenic *acrR<>acrAB* d region of the overlapping in three reactors (R1, R3, R5) reaching A_{max}~100% in one of them (R3).

MarR: Several intergenic mutational events were observed upstream of *marR* gene likely leading to derepression of *marRA* operon and, ultimately, to overexpression of AcrAB and TolC components of the *E. coli* efflux machinery. By extension, we consider a missense mutation MarR:Glu10Asp reaching A_{max}=28% in one of the reactors (R4) as potentially having a similar effect.

MdtK: We observed amplification of ~ 9 kB gene locus contains 2 genes upstream (*cfa* and *ribC*) and 6 genes downstream (ydhQ-W, mostly with unknown functions) of *mdtK* gene encoding a MATE family efflux transporter. Additional evidence implicating MdtK as a transporter contributing to efflux of GP-6 includes: (i) a detected intergenic mutation in *mdtK* upstream region potentially leading to upregulation of its expression; and (ii) similar genomic amplification of *A. baumannii* genomic locus harboring *mdtK* gene (see below) as the only common gene with the amplified locus in E. coli.

In A. baumannii (Figure S3B):

AdeIJK: Disruptive mutations (stop-gain, frameshift and IS insertions) in *adeN* gene encoding a repressor of a*deIJK* operon were observed in all 6 reactors rapidly expanding and sustaining at 100% aggregated abundance in 4 of them (R1-R4). Second, we observed two instances of ISAba1 insertions in the coding and intergenic regions of a gene (AUO97b_02717, a putative membrane-associated phospholipid phosphatase, PAP2-like) localized upstream of AdeIJK efflux pump encoding operon. They emerged at an early stage of evolution in 4 reactors (R1, R3, R5 and R6) reaching 65-100% abundance but sustaining in only two of them (R5 and R6) being competed out by AdeN:IS variants in the other two (R1 and R3). Of the two additional sporadic variants detected in the same locus (each at a single time point in a single reactor), a mutation in the intergenic region upstream of *adeIJK* operon may lead to its derepression (as in case of *adeN* disruptive mutations), while functional implications of a missense mutation His141Tyr in AUO97b_02717 (upstream of *adeIJK* operon) are unclear.

AdeAB: Two missense mutations in AdeS histidine kinase from AdeRS two-component system regulating AdeAB(C)[1] efflux pump were detected in R3. They emerged at the last stage of the evolution experiment reaching A_{max}=20% in case of the AdeS:Ser101Gly variant. Such variants were much more prominent in the previous CIP study [1], showing a broader repertoire of missense mutations (total of 7), more independent occurrences (total of 8) and higher A_{max}=65% (Table 1). Mutations in the *adeS* gene were previously observed as adaptation to antibiotic stress via AdeABC overexpression.

MdtK: We observed: (i) 3-11x amplification of ~11 Kb genomic locus containing *mdtK* gene in 5 out of 6 reactors*;* and (ii) insertion of *ISAba1* element in the gene immediately upstream of *mdtK* in all 6 reactors and reaching A_{max}~100% abundance in 5 of them; and (iii) deletion of ~2.5 kB genomic locus upstream of *mdtK* gene.

Other potentially significant variants.

<u>*In E. coli (Figure S3A)*</u>:

Rob: 3 mutational variants in the C-terminal domain of Rob transcriptional regulator: Rob:Ala201Thr, Rob:Gln194Pro and Rob:Cys147Tyr, the latter reaching 35% abundance.

RpoC: Gly336Ser variant (A_{max}=75% in R1) affecting β'-subunit of DNA-directed RNA polymerase complex (RNAP). While the impact of this mutation was not characterized, some mutational variants in RNAP β-subunit (RpoB) were implicated with CIP resistance and upregulation of *mdtK* gene [2]. The latter effect, if phenocopied by RpoC: Gly336Ser variant could indeed impact GP6 resistance.

Lon: An insertion of IS186B mobile element in the upstream noncoding region of the *lon* gene was detected in reactors R1 and R2, in the latter reaching A_{max}=77%.

YadG: A functional significance of a frameshift variant YadG:Val83fs (A_{max}=96% in reactor R5) is unlikely since, being a singleton, it may be just a "hitchhiker" reaching high abundance due to accidental coupling with a genuine driver mutation. Such interpretation would be consistent with the observation that R5 is fully dominated by a single GyrB:Thr165Ile variant (A_{max}=100%). Although this variant outcompeted another early-stage double mutant, GyrB:Thr165Ile/AcrR:Thr5Ile, it is more likely due to the *marRAB locus duplication also identified in this clone (see Supplementary Table S2A) than to the mutation in yadG gene per se.*

<u>*In A. baumannii (Figure S3B):*</u>

EttA: disruption of ettA gene encoding energy-dependent translational throttle A

---

[1] Note that many strains of *A. baumannii*, including ATCC17978 contain only two genes encoding AdeAB but not AdeC components of this, otherwise functionally active, efflux pump. The nature of the alternative third component is under investigation (*Dr. A. Kumar, personal communication*).

emerged in two reactors (R2 and R6) as two distinct variants, EttA:Asn239fs; EttA:ISAba. The latter variant reached A_{max}=76% in the last stage of evolution on the background of a triple mutant GyrB:Thr179Ile/AdeN:ISAba1/*us_mdtK*:ISAba1.

RplA: of the 3 distinct frameshift variants in the gene *rplA* encoding ribosomal protein L1, one variant RplA:Ile185fs reached A_{max}=76% in the background of a double mutant GyrB:Thr179Ile/AdeN:ISAba1.

MnmG: a low frequency (Amax=15%) in-frame insertion in *mnmG* gene (involved in tRNA modification) leading to a Val11-Ile12 duplication was observed in 4 reactors.

RpoB: two low frequency variants (A_{max}=10%) were observed in RNAP β-subunit, RpoB:Gln526Pro and in-frame insertion/replacement variant RpoB:Asn777_Val778ins[ThrCysIleAsnGlnAsn]. A potential significance of these relatively minor variants is supported by the already mentioned precedents of RpoB mutations [2] and, indirectly, by the emergence of RpoC:Gly336Ser variant during evolution of GP6 resistance in *E.coli*.

Supplementary Figure S4.

Genomic rearrangements leading to *mdtK* gene overexpression.

A: Amplifications (amp) and deletions (del) in genomic loci harboring *mdtK* multidrug efflux transporter gene detected in evolving populations (left) and in 5 selected clones (right) of *E. coli* and *A. baumanni* analyzed by RT-qPCR.

Genomic rearrangements in GP6 experimental evolution leading to MdtK overexpression								
Evolving populations samples (WGS)					Clones (WGS+Nanopore): RT-qPCR(MdtK-OE); MIC(GP6)			
Sample	Copy (Ave)	From	To	Type	Clone	Variant	OE Log2FC	MIC Log2FC
**in *E. coli***								
**1F**	~5x	1,691,374	1,747,336	Amp				
**2F**	~1x	-	-	WT	**2F4**	1x(MdtK:us_del)+Acr:IS	4.5	2
**3F**	~4.5x	1,726,732	1,734,752	Amp				
**4F**	~12x	1,734,868	1,743,493	Amp				
**5F**	~12x	1,736,523	1,746,503	Amp				
**6F**	~17x	1,721,962	1,741,383	Amp	**6F7**	15xMdtK+AcrR:T5N	2.8	4
(MdtK reference)		1,737,714	1,739,087	NA				
**in *A. baumannii***								
**1F**	~1x	-	-	WT	**1F1**	1x(MdtK:us_IS)+AdeN:IS	2.0	3
**2D**	~6x	1,521,679	1,534,791	Amp				
**3D**	~2.5x	1,521,635	1,550,459	Amp	**3D2**	9x(MdtK:us_IS)+AdeN:IS	5.9	5
**4F**	~0.05x	1,518,115	1,521,507	Del	**4F10**	1x(MdtK:us_del)+AdeN:E198*	5.5	5
**5F**	~7x	1,521,635	1,535,875	Amp				
**6F**	~5x	1,521,331	1,535,153	Amp				
(MdtK reference)		1,521,873	1,523,168	NA				

The extent of *mdtK* gene overexpression (OE) along with changes in MIC^{GP6} are expressed as $log_2(FC)$

B: Genomic rearrangements in the respective clones established by nanopore sequencing.

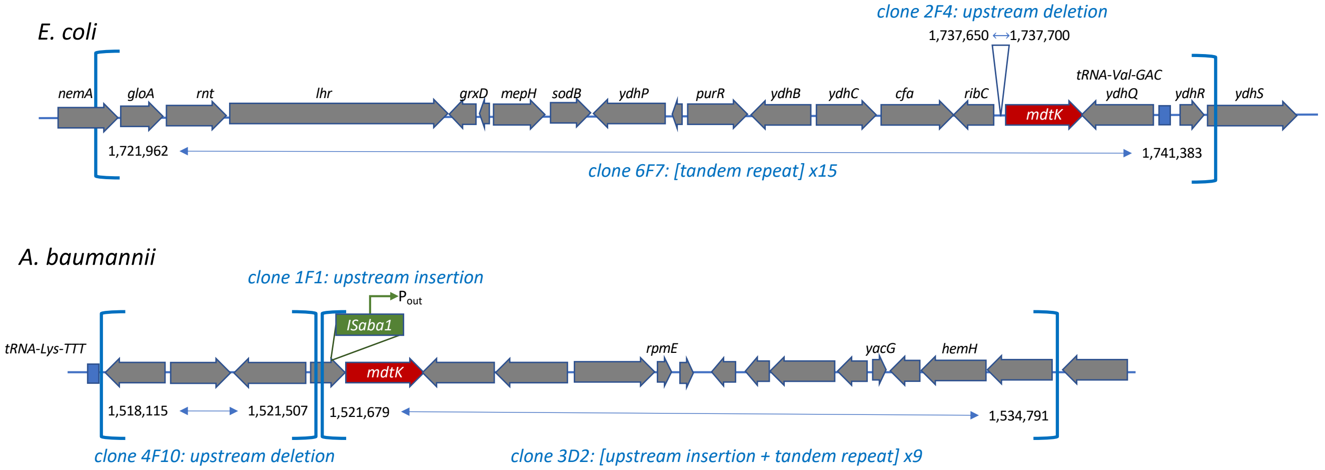

Supplementary Figure S4 (continued).

C: Testing of *mdtK* gene overexpression by RT-qPCR in selected *E. coli* clones harboring genomic rearrangements in *mdtK*-containing chromosomal locus: 50 bp upstream deletion (clone 2F4) or 15x locus amplification (clone 6F7). Clone 5C2 (carrying the same AcrR:IS5 variant as clone 2F4 but an intact *mdtK*-locus) and the unevolved parental strain (WT) are used as a negative control group.

(Details of the experimental and statistical analyses are provided in Supplementary Methods)

RT-qPCR analysis of mdtK expression in selected GP6-evolved *E. coli* clones vs WT

Sample	C_t (gyrB)	C_t (mdtK)	ΔC_t (mdtK-gyrB)	Average ΔC_t (mdtK-gyrB)	$\Delta\Delta C_t$ (relative to WT)	**log_2(FC) expression (realtive to WT**	**SD**	P-values one-way ANOVA with Tukey's HSD vs:		
								**2F4**	**5C2**	**6F7**
**2F4**	18.26	16.68	-1.58	-1.463	-4.54	**4.54**	0.15			
	17.75	16.45	-1.30							
	17.93	16.42	-1.51							
**5C2**	17.10	19.68	2.58	2.790	-0.29	**0.29**	0.19	1.0E-07	-	
	17.16	20.02	2.86							
	17.42	20.35	2.93							
**6F7**	17.55	17.79	0.24	0.303	-2.78	**2.78**	0.37	1.2E-04	9.6E-06	-
	18.04	18.01	-0.03							
	17.56	18.26	0.70							
**WT**	17.55	20.37	2.82	3.080	0.00	**0.00**	0.25	1.0E-07	5.3E-01	4.2E-06
	17.74	21.06	3.32							
	17.75	20.85	3.10							

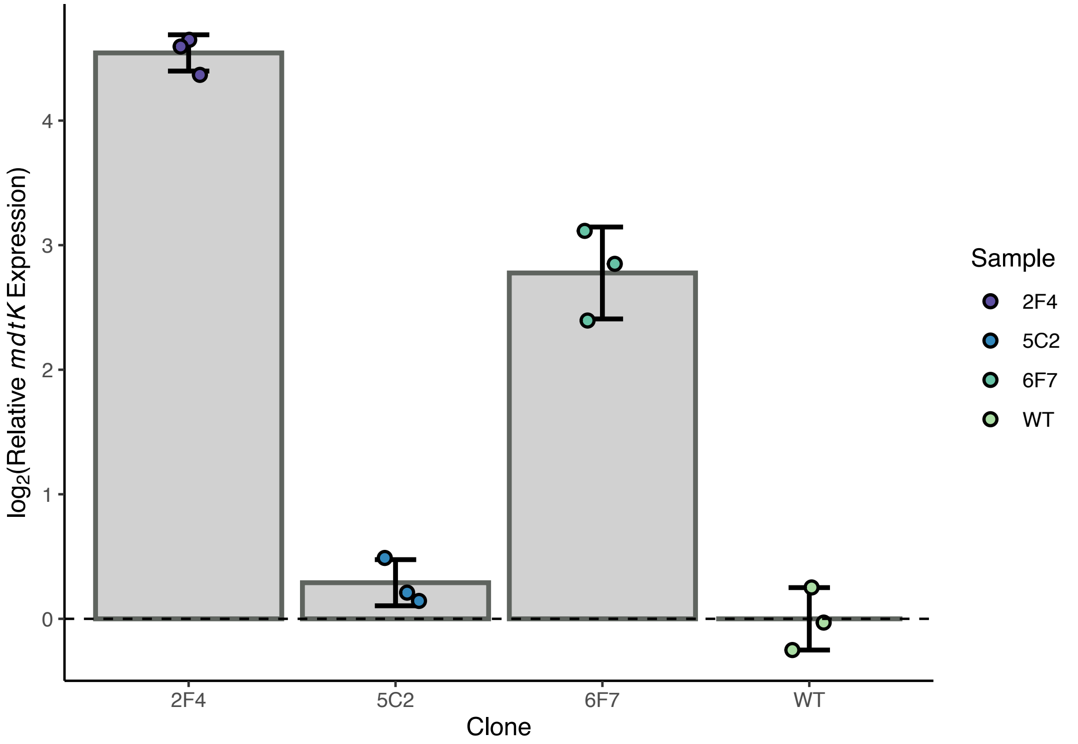

Supplementary Figure S4 (continued).

D: Testing of *mdtK* gene overexpression by RT-qPCR in selected *A. baumannii* clones harboring genomic rearrangements in *mdtK*-containing chromosomal locus: upstream IS-insertion (clone 1F1); upstream IS-insertion combined with 9x locus amplification (clone 2D2); or ~3.5 kB upstream deletion (clone 4F10). Clone 1F2 (carrying the same AdeN:IS variant as the clone 1F but an intact *mdtK*-locus) and the unevolved parental strain (WT) are used as a negative control group.
(Details of the experimental and statistical analyses are provided in Supplementary Metghods)

RT-qPCR analysis of mdtK expression in selected GP6-evolved *A. baumannii* clones vs WT

Sample	C_t (gyrB)	C_t (mdtK)	ΔC_t (mdtK-gyrB)	Average ΔC_t (mdtK-gyrB)	$\Delta\Delta C_t$ (relative to WT)	**$\log_2$(FC) expression (realtive to WT**	**SD**	P-values one-way ANOVA with Tukey's HSD vs:			
								**1F1**	**1F2**	**3D2**	**4F10**
**1F1**	19.1	19.58	0.48	-0.253	-1.96	**1.96**	0.64				
	18.59	17.93	-0.66								
	18.49	17.91	-0.58								
**1F2**	18.25	20.24	1.99	2.140	0.44	**-0.44**	0.18	4.0E-03	-		
	18.15	20.49	2.34								
	18.33	20.42	2.09								
**3D2**	18.44	13.71	-4.73	-4.195	-5.90	**5.90**	0.76	2.4E-04	4.9E-06	-	
	18.42	14.76	-3.66								
**4F10**	18.4	14.35	-4.05	-3.810	-5.51	**5.51**	0.67	2.2E-04	3.2E-06	9.4E-01	-
	18.33	15.28	-3.05								
	18.31	13.98	-4.33								
**WT**	18.55	20.56	2.01	1.703	0.00	**0.00**	0.52	1.4E-02	8.7E-01	8.9E-06	6.2E-06
	19.28	20.38	1.10								
	18.13	20.13	2.00								

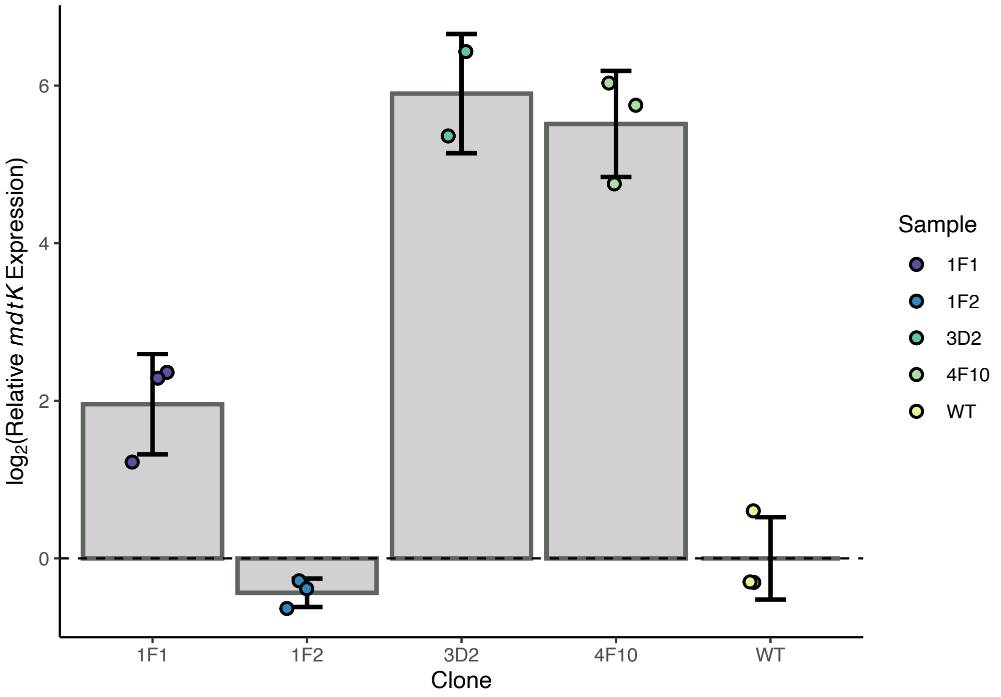

Supplementary Figure S5.

Structure of Rob transcriptional regulator complex with DNA (PDB:1D5Y [3]). Residues with variants observed in GP-6 morbidostat experiments are colored red.

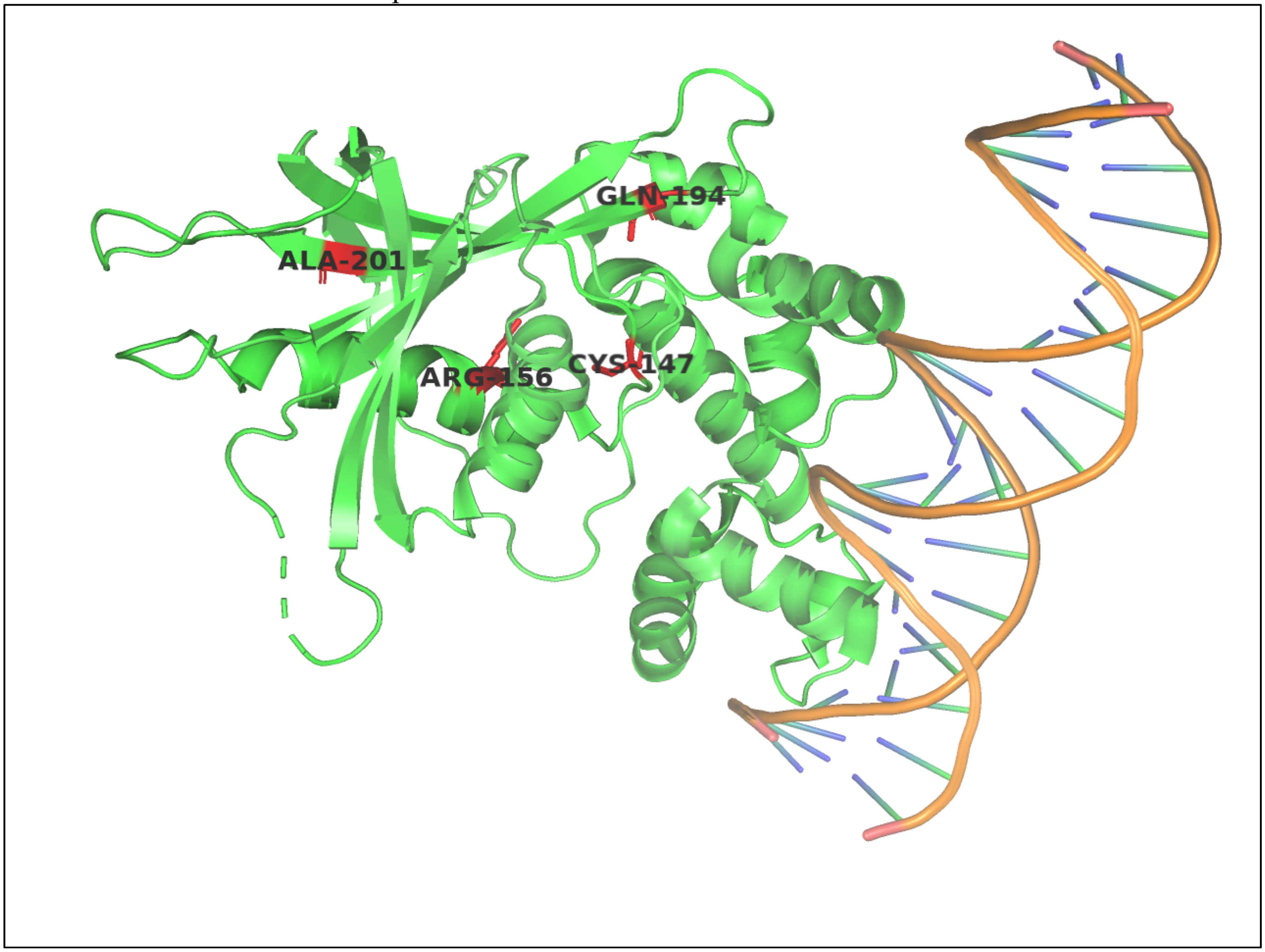

II. Supplementary Methods

Morbidostat setup and programming

Our implementation of the morbidostat approach was described in our previous studies with CIP and triclosan [1,4]. Briefly, the morbidostat is a computer-controlled chemostat-like continuous culturing bioreactor where density of bacterial culture is controlled by varying an antibiotic concertation in media. Automated dilutions switch between addition of; (i) drug-containing media (increase of a drug concentration) when bacterial cultures are growing faster than dilution rate, and (ii) drug-free media (decrease of a drug concentration) when the growth rate slows down due to excessive drug pressure. This algorithm enables a gradually growing selective pressure driving the evolution of higher drug resistance. The detailed description of morbidostat implementation is provided on GitHub (https://github.com/sleyn/morbidostat_construction). The main characteristic features of our implementation include:

1) Six glass culture tubes containing 20 mL of media are used as bioreactors equipped with stirring bars for culture agitation and three needles for: (a) input of media and air, (b) waste removal and (c) sample collection.
2) Laser beam intensity is used to measure culture turbidity (optical density or OD at ~635 nm). The laser, photodiode and culture tube are installed in the 3D-printed rack.
3) A miniature air pump provides aeration and maintains air pressure for liquid displacement fitted with 0.22 µm air filters for sterility.
4) Drug-containing media and drug-free media are fed from two bottles connected via autoclavable silicon tubing to computer-controlled peristaltic pumps, each for one media type, followed by an array of 12 valves (2 valves per reactor, one for controlling flow of drug-free media and one – for drug-containing media).
5) The excess volume from the reactors (upon each dilution cycle) media is displaced to waste bottles by the constant air flow.
6) The temperature in morbidostat chamber is kept at 37°C with a heater controlled by thermal sensor.
7) All logic operations are controlled by an Arduino Mega 2560 Rev3 board. A user interface is implemented on PC using MegunoLink software (v.1.32.20005.0105; https://www.megunolink.com/).

The program logics and parameters of automated dilutions were as follows:

1) Regular dilutions with one or the other media (20% by volume) are made over programmable time intervals (cycle time, CT).
2) To select a dilution mode, OD by the end of each cycle is compared with two thresholds: lower threshold (LT) and drug threshold (DT).
3) At LT ≤OD <DT (as in the beginning of the run or after growth deceleration upon reaching drug concentrations above the level of acquired resistance), dilutions are made with drug-free media using CT=15 min (which could be manually adjusted up to 20-25 min upon overall fitness loss at higher drug concentrations)
4) At OD ≥ DT, the selection of the media for every dilution cycle (CT=15 min by default) is made based on the following logics:
   - if $OD_1 \geq OD_0$ where OD_1 is OD at the end of the current cycle and OD_0 is OD at the end of the previous cycle (e.g. when the culture growth rate is faster than the dilution rate), the dilution is made by the drug-containing media;
   - Otherwise, (if $OD_1 < OD_0$, when the culture growth rate is slower than dilution rate), the dilution is made by the drug-free media.

5) At OD < LT (if it happens at poorly tolerable spike in drug concentration), the culture outgrowth was enabled via an automated increase of CT to 60 min with dilutions by the drug-free media (until reaching OD ≥LT).

Sequencing data analysis, variant calling and ranking

The raw reads were processed as previously described [1]. Briefly, upon adapter and quality trimming (BBDuk from BBTools suite v. 38.42, https://sourceforge.net/projects/bbmap/), the reads were aligned to reference genomes (BWA MEM v0.7.17 [5]). LoFreq Viterbi module was used to refine alignment near indel regions [6]. Base Quality Score Recalibration was made by Genome Analysis Toolkit (GATK) modules BaseRecalibrator and ApplyBQSR [7]. Sites with high frequency variants were masked from BaseRecalibrator by calling variants in the alignment down-sampled to ~ 50x coverage with Picard tools v.2.2.1 (https://broadinstitute.github.io/picard/) based on the coverage estimates by mosdepth v. 0.2.6 [8]. The VCF file for BaseRecalibrator "--known_sites" option was produced by GATK HaplotypeCaller. All other SAM and BAM files manipulations (sorting, indexing, merging and splitting) were performed with Samtools v1.9 [5]. SNP and indels were identified using LoFreq v2.1.3 [6].

Insertion sequence (IS) elements rearrangements were identified by a developed iJump tool (https://github.com/sleyn/ijump). IS elements in the reference genomes were predicted using BLAST against ISFinder database [9]. Predicted effects of mutations were assigned with SnpEff v4.3 [10]. VCF files manipulations were performed with bcftools v1.3 [11]. Copy number variation (CNV) were predicted by CNOGPro package v1.1 for R [12]. Reference genomes were downloaded from PATRIC database [13]. Statistics of read alignments against reference genomes is in Supplementary Table S1C for population samples and is in Supplementary Table S2C for clone samples. Reference genome IDs are 679895.18 for *E. coli* BW25113 and 400667.82 for *A. baumannii* ATCC17978. WGS data and assembly of all six unevolved clones (A1 – A6) including variations compared to reference genomes were reported in the previous study [1]. Nanopore reads were base-called using Guppy v.5.0.16 and aligned to reference with minimap2 v.2.17 [14]. Large rearrangements were verified using Ribbon tool [15].

Ranking of the observed variants as significant was performed based on their statistics across all six reactors as described [1] and illustrated in Supplementary Tables S1A and B that provide complete lists of observed variants combing SNVs, short indels, IS inserts and CNV data. Their ranking is reflected in the column "Category" of both tables, which is used to apply filters limiting the display to highly ranked (potential drivers and strongly coupled mutational events). Our approach to ranking, after exclusion of technically unreliable calls (frequency <2%, imperfect repeats, etc.) as well as "preexisting" variants and synonymous mutations was gene-centered based on two major (complementary) features. First, we prioritize variants reaching high frequency (or maximal abundance, in a population sample weighted on the total site abundance, $\boldsymbol{A_{max}}$) in at least one sample across all 6 reactors. Then, for all genes implicated by at least one variant with $\boldsymbol{A_{max}}$ ≥10% (coding sequences and their upstream noncoding regions, and, in case of IS-inserts or large deletions, including upstream coding regions) we consider all variants (non-preexisting, non-synonymous and with $\boldsymbol{A_{max}}$≥2%). The second feature for further ranking of these initially prioritized genes, reflects the overall number of independently occurring variants per gene ($\boldsymbol{N_{all}}$). The latter combines a total number of distinct variants (***V***) with the number of reactors where each variant independently arises (***R***). Of those, the first parameter (***V***) is typically more significant, especially for disruptive mutations in coding regions (such as frameshifts, stop-gain and IS inserts). Therefore, we additionally prioritize the genes with high values of V>3, even with 2%≤$\boldsymbol{A_{max}}$<10% for all distinct variants, as long as their total abundance exceeds 10% in at least one sample. This approach typically implicates up to 8-10 genes with $\boldsymbol{A_{max}}$≥50% and N_{all}≥3 (up to 20 for disruptive mutations) as illustrated in Table 1. A functional significance of up to 5-6 genes with lower rank (smaller values of one or both parameters) remains questionable, and, typically, not supported by clonal analysis. In contrast to SNVs and even IS-inserts (where abundance determination is intrinsically less accurate), CNVs (large deletions

and amplifications) cannot be accurately estimated in population WGS data. Besides, they cannot be unambiguously assigned to a specific gene, as typically cover from 3 to more than 30 genes (see Supplementary Tables S1A and B). Therefore, the prioritization (and interpretation) of the observed CNV events relies on the comparison with a list of genes implicated by other types of events (as for *mdtK* and *marR* genes in this study). Identification of mutations in clones is straightforward. The observed variants typically match those from respective population data (although in rare cases additional mutations are observed), and their calculated abundance is usually >90% (see Supplementary Tables S2A and B).

Applying these criteria to both WGS datasets allowed us to define the list of significant variants (excluding CNVs): (i) for *E. coli*, 34 distinct events in 14 genes; and (ii) for *A. baumannii*, 30 distinct events in 10 genes (displayed in the **Supplementary Tables S1A and B** after applying respective filters).

Population WGS data deconvolution for evolutionary dynamics and clonal analysis

The overall abundance of all (even highly ranked) mutations in a sample commonly exceeds 100%, especially at the later stages of experimental evolution pointing to the emergence of double and triple mutants (more characteristic of *A. baumannii* than *E. coli*, see Supplementary Figure 3). Although, the accurate assignment of these mutations to clonal subpopulations in many cases is nearly impossible, we used an approximate deconvolution of population samples for assessment of trends in evolutionary dynamics and optimization of clone isolation strategy aimed to maximize the coverage of the major mutational variants and combinations thereof.

We used Mixed Integer Programming to deconvolute population sequencing data represented by a matrix of variant frequencies (***F***) where rows are variants, columns are sampling time points and values represent variant frequencies in the population raging from 0 to 100% (as in *Supplementary Tables S1 A and B*). Since population samples are composed of one or more competing subpopulations that emerge and expand or wash out, each variant frequency (***f***) is a sum of frequencies of subpopulations where this variant is present:

$$f_n^k = \sum_{i=1}^{m} s_i^k p_{ni}^k$$

where f_n^k is a frequency of an *n*-th observed variant in a *k*-th sample; s_i^k – frequency of an *i*-th subpopulation in the *k*-th sample, p_{ni}^k – a dummy variable, which is equal to 1 if the *n*-th variant *n* is present or 0 if it is absent in the *i*-th subpopulation in the *k*-th sample. Then the matrix of variant frequencies ***F*** can be computed as matrix multiplication:

$$P \cdot S = F$$

where *P* is a binary matrix of dummy variables with rows representing variants and columns representing subpopulations; and *S* – a matrix of subpopulation frequencies with rows representing subpopulations and columns representing population samples. The deconvolution of populations is formalized as an optimization problem to find values of matrices *P* and *S* given the following constrains:

1) Values of matrix *P* are binary.
2) Values of matrix *S* have boundaries 0 and 100%.
3) Sum of each column of matrix *S* should be within the range from 0 to 100%.

The algorithm was implemented using PuLP toolkit of Python 3 [16] and Gurobi solver [17]. The details of the algorithm and its implementation could be found at the GitHub page (https://github.com/sleyn/subpop_decompose_mip). The obtained subpopulation frequencies were used to choose the samples with the largest representation of the most prominent subpopulations (with s≥20%)

that would allow us to maximize the odds and minimize the redundancy in their representation in clonal analysis.

RNA isolation and RT-qPCR

RNA isolation from flash-frozen pellets was performed as described using a modified protocol of [18] with minor modifications. Briefly, cell pellets were resuspended in lysis buffer (NaCl, 140 mM; EDTA, 14 mM; SDS, 6 % w/v) and mixed with Phenol_Chloroform: Iodoacetic acid (125: 24: 1, pH 4.5). Glass beads (acid washed, 500 uL) were added to each sample before being homogenized using a Bead Ruptor 12 (Omni Inc) for 2 minutes at 6 m/s 2 times. Subsequently, the samples were centrifuged (15 minutes, 16000 *g*, 4 °C) and the aqueous phase collected. Crude RNA was precipitated from this phase using sodium acetate (0.3 M final concentration) and isopropanol overnight at -20 °C. The precipitates from overnight were collected by centrifugation (30 minutes, 16000 *g*, 4 °C), washed 2 times in ice cold ethanol (70 %), dried in a laminar flow hood, and resuspended in nuclease free water. Coprecipitated DNA was removed using Baseline-ZERO™ DNase (Lucigen) following the manufacturers protocol. Samples were further purified using the MEGAclear™ Transcription Clean-Up Kit (Invitrogen). RNA concentration and quality was assessed via both nanodrop, using A260/280 and A260/230 ratios, and 1 % TAE agarose gel to monitor ribosomal RNA integrity. For samples which exhibited poor A260/A230 ratios, an additional round of ethanol precipitation was performed using the GlycoBlue™ coprecipitant kit (Invitrogen), following the manufacturers instructions. Isolated RNA was frozen in liquid nitrogen and stored at -80 °C.

Complementary DNA was prepared from the isolated RNA using the High Capacity cDNA Reverse Transcription Kit (Thermo Fisher Scientific) following the manufacturers direction, with 200 ng of cDNA being prepared per clone per replicate. qPCR reactions were prepared using the PowerTrack™ SYBR™ Green Master Mix (Thermo Fisher Scientific) with 10 ng of prepared cDNA being used per gene for each sample and a final primer concentration of 400 nM. The reactions were performed on a Bio-Rad CFX384 Touch Real-Time PCR System with the following conditions: 95 °C for 10 minutes; 40 cycles of 95 °C for 15 s, 55 °C for 60 s. Threshold cycle (Ct) values were assigned for both the target gene, *mdtK*, and the reference gene, *gyrB* within CFX Manager™ (Bio-Rad). Relative expression of *mdtK* and the associated standard deviations calculated for each clone using the $2^{-\Delta\Delta CT}$ method [19]. One-way ANOVA with Tukey's HSD post-hoc testing was performed in R. The primers used are shown below.

For *A. baumannii*

Target for the assessment of overexpression

mdtK FW primer: ATTCCAGTGATTGTCCGCCA

mdtk RV primer: ATGCTTCGGAATAGCCTCGG

Housekeeping control for normalization[20]

gyrB FW primer: GATGATGCGCGTGAAGGTTT

gyrB RV primer: CATTGCTTGCTCTACCGCTG

For *E. coli*

Target for the assessment of overexpression

mdtK FW CTCTTTGGTCACGGACTGCT

mdtk RV TACGGGCAACCTGGAAGAAC

Housekeeping control for normalization

gyrB FW GAACAAAACGCCGATCCACC
gyrB RV GCACTTTCACGGAAACGACC

REFERENCES FOR THIS DOCUMENT

1 Zlamal, J. E. *et al.* Shared and Unique Evolutionary Trajectories to Ciprofloxacin Resistance in Gram-Negative Bacterial Pathogens. *mBio* **12**, e0098721 (2021). https://doi.org:10.1128/mBio.00987-21

2 Pietsch, F. *et al.* Ciprofloxacin selects for RNA polymerase mutations with pleiotropic antibiotic resistance effects. *J Antimicrob Chemother* **72**, 75-84 (2017). https://doi.org:10.1093/jac/dkw364

3 Kwon, H. J., Bennik, M. H., Demple, B. & Ellenberger, T. Crystal structure of the Escherichia coli Rob transcription factor in complex with DNA. *Nat Struct Biol* **7**, 424-430 (2000). https://doi.org:10.1038/75213

4 Leyn, S. A. *et al.* Experimental evolution in morbidostat reveals converging genomic trajectories on the path to triclosan resistance. *Microb Genom* **7** (2021). https://doi.org:10.1099/mgen.0.000553

5 Li, H. & Durbin, R. Fast and accurate short read alignment with Burrows-Wheeler transform. *Bioinformatics* **25**, 1754-1760 (2009). https://doi.org:10.1093/bioinformatics/btp324

6 Wilm, A. *et al.* LoFreq: a sequence-quality aware, ultra-sensitive variant caller for uncovering cell-population heterogeneity from high-throughput sequencing datasets. *Nucleic Acids Res* **40**, 11189-11201 (2012). https://doi.org:10.1093/nar/gks918

7 DePristo, M. A. *et al.* A framework for variation discovery and genotyping using next-generation DNA sequencing data. *Nat Genet* **43**, 491-498 (2011). https://doi.org:10.1038/ng.806

8 Pedersen, B. S. & Quinlan, A. R. Mosdepth: quick coverage calculation for genomes and exomes. *Bioinformatics* **34**, 867-868 (2018). https://doi.org:10.1093/bioinformatics/btx699

9 Siguier, P., Perochon, J., Lestrade, L., Mahillon, J. & Chandler, M. ISfinder: the reference centre for bacterial insertion sequences. *Nucleic Acids Res* **34**, D32-36 (2006). https://doi.org:10.1093/nar/gkj014

10 Cingolani, P. *et al.* A program for annotating and predicting the effects of single nucleotide polymorphisms, SnpEff: SNPs in the genome of Drosophila melanogaster strain w1118; iso-2; iso-3. *Fly (Austin)* **6**, 80-92 (2012). https://doi.org:10.4161/fly.19695

11 Li, H. A statistical framework for SNP calling, mutation discovery, association mapping and population genetical parameter estimation from sequencing data. *Bioinformatics* **27**, 2987-2993 (2011). https://doi.org:10.1093/bioinformatics/btr509

12 Brynildsrud, O., Snipen, L. G. & Bohlin, J. CNOGpro: detection and quantification of CNVs in prokaryotic whole-genome sequencing data. *Bioinformatics* **31**, 1708-1715 (2015). https://doi.org:10.1093/bioinformatics/btv070

13 Davis, J. J. *et al.* The PATRIC Bioinformatics Resource Center: expanding data and analysis capabilities. *Nucleic Acids Res* **48**, D606-D612 (2020). https://doi.org:10.1093/nar/gkz943

14 Li, H. Minimap2: pairwise alignment for nucleotide sequences. *Bioinformatics* **34**, 3094-3100 (2018). https://doi.org:10.1093/bioinformatics/bty191

15 Nattestad, M., Aboukhalil, R., Chin, C. S. & Schatz, M. C. Ribbon: intuitive visualization for complex genomic variation. *Bioinformatics* **37**, 413-415 (2021). https://doi.org:10.1093/bioinformatics/btaa680

16 Mitchell, S., O'Sullivan, M. J. & Dunning, I.

17 Gurobi Optimizer Reference Manual} (2022).

18 Rey, F. E. *et al.* Dissecting the in vivo metabolic potential of two human gut acetogens. *J Biol Chem* **285**, 22082-22090 (2010). https://doi.org:10.1074/jbc.M110.117713

19 Schmittgen, T. D. & Livak, K. J. Analyzing real-time PCR data by the comparative C(T) method. *Nat Protoc* **3**, 1101-1108 (2008). https://doi.org:10.1038/nprot.2008.73

20 Wong, M. H., Chan, B. K., Chan, E. W. & Chen, S. Over-Expression of ISAba1-Linked Intrinsic and Exogenously Acquired OXA Type Carbapenem-Hydrolyzing-Class D-ss-Lactamase-Encoding Genes Is Key Mechanism Underlying Carbapenem Resistance in Acinetobacter baumannii. *Front Microbiol* **10**, 2809 (2019). https://doi.org:10.3389/fmicb.2019.02809
